# Measurement of PD-L1 in melanoma: a quantitative antibody comparison

**DOI:** 10.1186/2051-1426-3-S2-P107

**Published:** 2015-11-04

**Authors:** Joel C Sunshine, Sneha Berry, Jessica Esandrio, Haiying Xu, Aleksandra Ogurtsova, Toby Cornish, Evan J Lipson, Robert A Anders, Janis M Taube

**Affiliations:** 1 Johns Hopkins University School of Medicine, Baltimore, MD, USA; 2 Sidney Kimmel Comprehensive Cancer Center, Baltimore, MD, USA

## Background

Immunohistochemical detection of PD-L1 may be used in the future as a biomarker to help select an immunotherapeutic regimen for patients with advanced melanoma. For example, patients whose tumors are PD-L1+ may receive anti-PD-1 monotherapy, and those whose tumors are PD-L1(-) may receive combination anti-PD-1 and anti-CTLA-4. The evaluation of the utility of PD-L1 as a biomarker has been hampered by the different antibodies and assays used. The purpose of this study was to quantitatively compare staining properties of three different PD-L1 monoclonal antibodies that have been used in recent landmark publications.

## Methods

Immunohistochemistry for PD-L1 was performed on serial sections from fourteen formalin-fixed paraffin-embedded archival melanoma samples using three different monoclonal antibodies: 5h1, SP142, and E1L3N. Slides were imaged using the Vectra Automated Quantitative Pathology Imaging System, and using intensity thresholds set by two pathologists, were scored for the percentage of total cells (melanocytes and immune cells) demonstrating PD-L1 staining. The observed staining intensity in the samples below the threshold considered to be “positive” by the pathologists was used as a measure of background staining for each antibody.

## Results

**Figure 1:**
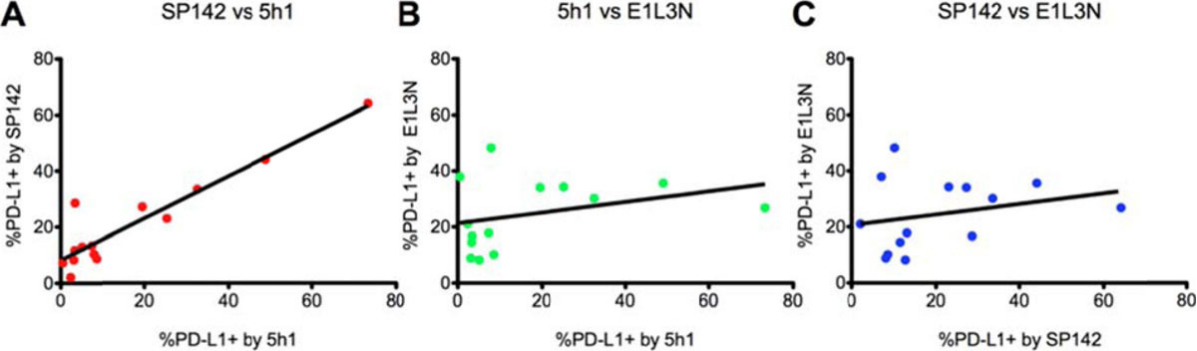


Membranous PD-L1 expression in melanoma samples detected with 5h1 and SP142 was highly correlated (Figure 1A, R2 = 0.88, p < 0.0001). However, there was no significant correlation between the results obtained with E1L3N vs 5h1 (Figure 1B, R^2^ = 0.11, p = 0.25) or SP142 (Figure 1C, R2 = 0.07, p = 0.36). The best fit line predicts that a specimen stained with 5h1, which scored as having 5% membranous PD-L1 expression, would show 12% PD-L1+ cells (95% confidence interval of 7.5% to 16.1%) when stained with SP142. 5h1 showed the lowest background staining, with the lowest intensity of staining in cells that were considered by the pathologists to represent PD-L1-negative cells. SP142 demonstrated staining properties very close to 5h1, but with slightly higher background staining intensity. E1L3N showed a higher background staining intensity than either of the other two clones, resulting in a less evident discrimination between PD-L1+ vs. PD-L1(-) cells.

## Conclusions

The commercially available clone SP142 demonstrates comparable staining properties to 5h1, while the E1L3N, at least under the conditions used in this study, does not. Similar comparisons including additional proprietary clones, such as 28-8 and 22C3, will be essential in understanding how best to use reported PD-L1 status from various assays to guide therapeutic selection in patients with advanced melanoma.

